# Factors Associating with Non-Dipping Pattern of Nocturnal Blood Pressure in Patients with Essential Hypertension

**DOI:** 10.3390/jcm12020570

**Published:** 2023-01-10

**Authors:** Tsutomu Koike, Teruhiko Imamura, Fumihiro Tomoda, Maiko Ohara, Hayato Fujioka, Kota Kakeshita, Hidenori Yamazaki, Koichiro Kinugawa

**Affiliations:** 1 Second Department of Internal Medicine, University of Toyama, 2630 Sugitani, Toyama 930-0194, Japan; 2 Faculty of Health Science, Fukui Health Science University, Fukui 910-3190, Japan

**Keywords:** hypertension, hemodynamics, oxidative stress

## Abstract

Background: In patients with essential hypertension, a non-dipping blood pressure pattern is a strong risk factor for cardiovascular diseases. However, background factors associating with such a blood pressure pattern remain unknown. Methods: Untreated essential hypertensive patients without chronic kidney diseases who were admitted to our outpatient clinic were included. Blood sampling and 24 h ambulatory blood pressure monitoring were mandatorily performed. Non-dipper status was defined as a maximum decrease in nocturnal systolic blood pressure within 10%. Clinical factors associating with non-dipper status were investigated. Results: A total of 154 patients (56 ± 12 years old, 86 men) were included. Among baseline characteristics, a higher serum uric acid level was independently associated with non-dipper status (odds ratio 1.03, 95% confidence interval 1.00–1.05, *p* < 0.05). Among those with non-dipper status, a higher high-sensitivity C-reactive protein level tended to be associated with incremental nighttime systolic blood pressure levels (*p* = 0.065). Conclusions: Hyperuricemia and micro-inflammation might be associated with attenuated nocturnal blood pressure dipping and incremental nighttime systolic blood pressure levels.

## 1. Background

Hypertension is one of the most common and major chronic diseases all over the world, and one of the most predominant risk factors for cardiovascular complications [1]. Of note, 24 h ambulatory blood pressure monitoring (ABPM) is superior to office blood pressure in predicting clinical outcomes [2,3]. Furthermore, by analyzing detailed diurnal variation, a non-dipper status, which is defined as a loss of physiological decline in blood pressure during nighttime, has a more considerable impact on end-organ injury and the occurrence of cardiovascular events compared with those with a dipper status [4,5,6,7,8].

The detailed causality and mechanism of the development of non-dipper status remain uncertain. Although few studies have been reported, chronic inflammation, of which serum uric acid level is one of the indicators, might be associated with the progression of non-dipping of nocturnal blood pressure [9,10]. Such investigations might clarify the etiologies of non-dipper status and let us construct therapeutic strategies targeting the etiologies to treat hypertension with non-dipper phenotype. In this study, we investigated factors associated with non-dipper status among those with newly diagnosed essential hypertension.

## 2. Methods

### 2.1. Patient Selection

Consecutive patients with untreated essential hypertension who were admitted to our outpatient clinic for the first time between January 2004 and December 2015 were retrospectively included. None of the patients had ever been admitted to any clinics to treat their hypertension beforehand. Blood pressures were measured in the interview room using automatic sphygmomanometers (ES-H55, Terumo Corporation, Tokyo, Japan) several times at rest-sitting condition, according to the Japanese Society of Hypertension Guidelines for the Management of Hypertension [11]. Two stable measurements were averaged and defined as a clinic blood pressure. Essential hypertension was defined as clinic blood pressure ≥140/90 mmHg following the exclusion of secondary hypertension.

Patients with chronic kidney disease (CKD), which was defined as an estimated glomerular filtration ratio <60 mL/min/1.73 m^2^ or urinary albumin excretion ≥300 mg/day, were excluded. Patients receiving anti-hyperuricemic agents were also excluded.

The present study was approved by the institutional review board. The informed consents were wavered given the retrospective nature of this study and the opt-out of this study protocol. The study was conducted in accordance with the Declaration of Helsinki.

### 2.2. Collected Data

Laboratory data were obtained from blood and urine samples on the same day of ambulatory 24 h blood pressure monitoring (ABPM) prior to the initiation of anti-hypertension treatment. Baseline demographics data, including laboratory and echocardiography data, were retrieved on the same day. Nitric oxide metabolites were measured by Griess test. The homeostasis model assessment of insulin resistance was calculated as follows: plasma fasting glucose (mg/dL) × immunoreactive insulin (μU/mL)/405. Serum leptin and adiponectin were measured by using an enzyme-linked immunosorbent assay kit.

24 h ABPM was performed using a Takeda TM-2431 (A&D Medical, Tokyo, Japan). On 24 h ABPM, blood pressure was recorded at 30-min intervals. The following parameters were calculated from 24 h ABPM: the average of systolic and diastolic blood pressure during 24 h, daytime (9:00 AM to 9:00 PM) and nighttime (0:00 AM to 5:00 AM). If >30% of the measurements were errors and missing, such cases were eliminated from the analyses.

**Figure 1:**
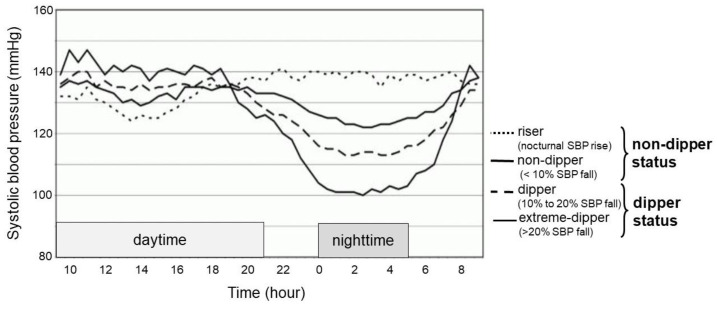
Classification according to circadian blood pressure pattern. According to ambulatory 24 h blood pressure monitoring, non-dipper hypertension was defined as a decrease in nocturnal systolic blood pressure <10%. SBP, systolic blood pressure.

The percentage of nocturnal systolic blood pressure decline was calculated as follows: (daytime average systolic blood pressure − nighttime average systolic blood pressure) × 100/daytime average systolic blood pressure. According to the 24 h ABPM, non-dipper hypertension was defined as a decrease in nocturnal systolic blood pressure within 10% (Figure 1).

### 2.3. Primary and Secondary Concerns

A primary concern was the factors associated with the non-dipper status. A second concern was the factors associated with nighttime average systolic blood pressure among those with non-dipper status.

### 2.4. Statistical Analyses

Continuous variables are stated as mean and standard deviation. Categorical variables are stated as numbers and percentage. Continuous variables were compared between the two groups using an unpaired t-test. Categorical variables were compared between the two groups using the Fisher’s exact test.

Factors associated with the existence of non-dipper status were investigated using logistic regression analyses among potential clinical parameters (aging, sex, excess sodium intake, sympathetic nervous system over activity, renin-angiotensin system, obesity, hyperinsulinemia, endothelial dysfunction, inflammation and hyperuricemia). Among those with non-dipper status, factors associated with nighttime systolic blood pressure were investigated using linear regression analyses among potential clinical parameters. In both regression analyses, variables with *p* < 0.10 in the univariable analyses were included in the multivariable analyses with frothed method following the confirmation of their variance inflation factors <10.

Before conducting this retrospective study, we analyzed the required sample size for the primary statistics. We estimated the odds ratio of 2, alpha error of 0.05, and 1-beta value of 0.80. The required sample size was calculated as 113.

Two-sided *p*-values < 0.05 were considered statistically significant. Analyses were performed using R software version 3.5.2 (R Foundation for Statistical Computing, Vienna, Austria).

## 3. Results

### 3.1. Baseline Characteristics

A total of 154 patients were included. Mean age was 55.7 ± 11.9 years old and 88 (57%) were men. No patients had active infection, malignancy, collagen disease, and cardiovascular diseases. Two patients had type 2 diabetes mellitus and 11 patients had dyslipidemia. The duration of hypertension was 4.9 ± 6.7 years. Mean 24 h systolic blood pressure was 142 ± 16 mmHg. Baseline demographics, laboratory data, and urinary data were summarized in Table 1.

There were 56 patients (36%) with non-dipper status. Patients with non-dipper status were characterized by elevated average systolic blood pressure during 24 h and nighttime, increased body mass index, serum uric acid, and high-sensitivity C-reactive protein (CRP), and decreased plasma nitric oxide metabolites compared with those with dipper status. The amount of alcohol intake was not statistically different between the two groups.

### 3.2. Factors Associated with Non-Dipper Status

Among laboratory data, uric acid, high-sensitivity CRP, and low-density lipoprotein cholesterol were higher, and high-density lipoprotein cholesterol and nitric oxidative metabolites were lower in the non-dipper status than the dipper status (*p* < 0.05 for all; Table 1). Urinary variables were not significantly different between those with and without non-dipper status (*p* > 0.05 for all).

Among potential clinical parameters, uric acid and high-sensitivity CRP, as well as body mass index, were associated with the existence of non-dipper status (*p* < 0.05 for all; Table 2). Of them, only uric acid was an independent determinant of non-dipper status with an adjusted odds ratio of 1.03 (95% confidence interval 1.00–1.05, *p* < 0.05).

### 3.3. Factors Associated with Systolic Blood Pressure among Those with Non-Dipper Status

Among potential baseline characteristics, high-sensitivity CRP tended to be associated with nighttime average systolic blood pressure in the multivariable analysis (*p* = 0.065; Table 3).

**Figure 2:**
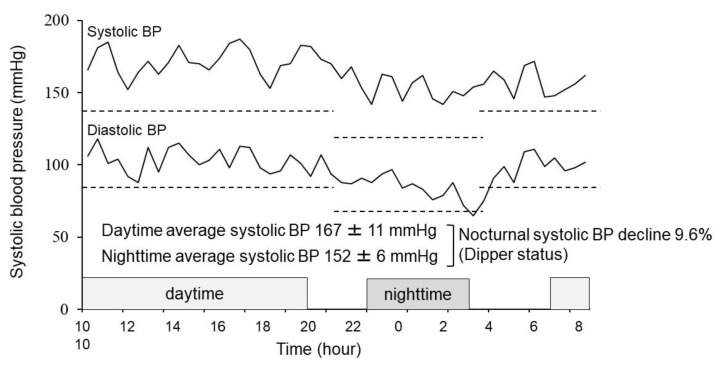
A representative patient with non-dipper status.

There were no statistically significant determinants of nighttime average systolic blood pressure in the dipper cohort (*p* > 0.05 for all). We display here a representative patient with non-dipper status, presenting 8.2 mg/dL of uric acid, 0.175 mg/dL of high-sensitivity CRP (Figure 2).

A 54-year-old woman with non-dipper status with a nocturnal systolic blood pressure decline = 9.6%, presenting 8.2 mg/dL of uric acid, 0.175 mg/dL of high-sensitivity CRP. BP, blood pressure.

## 4. Discussion

This study investigated factors associated with non-dipper status in patients with essential hypertension. Hyperuricemia was independently associated with non-dipper status. Among those with non-dipper status, high-sensitivity CRP tended to be associated with incremental nighttime average systolic blood pressure.

### 4.1. Hyperuricemia and Non-Dipper Status

Patients with essential hypertension often have comorbid hyperuricemia [12], which is an independent risk factor for cardiovascular events [13].

In patients with hypertension, the stimulated renin-angiotensin system and sympathetic nerve system both decrease the blood flow of renal medulla, accumulating serum uric acid levels. Furthermore, when activated, these systems stimulate reabsorption of uric acid via the urate transporter 1, which is located on the proximal tubule [14]. Thus, the excretion of uric acid is restricted in patients with hypertension.

In another study, the serum uric acid level was higher in those with non-dipper status compared with those with dipper status [15,16], which is consistent with our findings. Persistent nocturnal hypertension without any dippers might be caused by periodic hypoxia and stimulation of the sympathetic nerve system [17], all of which would further decline the excretion of uric acid via the above-described mechanisms.

On the contrary, it is unclear whether hyperuricemia itself would contribute to the development of hypertension. Some experimental studies showed controversial findings [18,19]. Others demonstrated that hyperuricemia would facilitate atherosclerosis via stimulation of inflammatory and oxidative stress systems [20,21].

We can at least hypothesize that the existence of hyperuricemia would be a surrogate of non-dipper status. Strict management of 24 h blood pressure might be encouraged if we find such patients. Whether intervention to hyperuricemia would ameliorate non-dipper status remains uncertain.

### 4.2. Chronic Inflammation and Further Incremental Blood Pressure

Among those with non-dipper status, some patients had further incremental 24 h blood pressure. An elevated high-sensitivity CRP was independently associated with it [9,10].

High-sensitivity CRP is one of the major indexes of chronic inflammation, which impairs endothelial function and facilitates microvasculature remodeling. Progression of microvasculature remodeling increases vascular resistance and causes essential hypertension [22]. The level of serum CRP in the healthy cohort is a risk factor for future essential hypertension [23].

According to experimental studies, hyperuricemia stimulates the release of various cytokines [24,25]. The serum uric acid level was associated with several inflammatory markers in the healthy cohort study [26]. Activated inflammatory status might be the next therapeutic target in patients with non-dipper status and refractory hypertension.

### 4.3. Limitations

Some comparisons might not reach statistical significance given the moderate sample size. We used the expected odds ratio of 2.0 to estimate the required sample size. However, given the lack of any similar studies, we cannot show robust references to validate our estimation. Of note, obesity and nitric oxide might also have associations with non-dipper status, although their impacts did not reach statistical significance. We performed 24 h ABPM only once per patient. We assessed the association among variables and causality remains uncertain. We did not measure several potentially important clinical factors, including the existence of sleep apnea and waist circumference, which might also affect our findings. We excluded those with CKD, given that CKD is a well-known strong contributor of non-dipping pressure pattern [27]. We hypothesized that the existence of CKD might be a considerable potential confounder that affects the impacts of other variables on the primary outcome. We also included extreme-dipper in the dipper group (normal group). An extreme-dipper might rather be associated with worse clinical outcomes [28].

## 5. Conclusions

Hyperuricemia and micro-inflammation might be associated with attenuated nocturnal blood pressure dipping and incremental nighttime systolic blood pressure levels. Their detailed causality and associated therapeutic strategy remain the next concern.

## Figures and Tables

**Table 1:** Baseline characteristics.

	Total (*n* = 154)	Non-Dipper (*n* = 56)	Dipper (*n* = 98)
Demographics			
Age (years)	55.7 ± 11.9	57.8 ± 12.7	54.5 ± 11.2
Male (number, %)	88 (57)	33 (59)	55 (56)
Duration of hypertension (years)	4.9 ± 6.7	5.6 ± 7.6	4.4 ± 6.1
Smoking (number, %)	41 (27)	16 (29)	25 (26)
Alcohol intake (g ethanol/day)	13.4 ± 19.8	14.3 ± 22.5	12.8 ± 18.3
Type 2 diabetes mellitus (number, %)	2 (1)	1 (2)	0 (0)
Dyslipidemia (number, %)	11 (7)	6 (11)	5 (5)
Body mass index (kg/m^2^)	24.6 ± 3.9	25.5 ± 4.2 *	24.1 ± 3.6
Blood Pressure			
24 h systolic BP (mmHg)	142 ± 16	148 ± 17 *	139 ± 16
24 h diastolic BP (mmHg)	88 ± 11	89 ± 10	88 ± 12
Daytime average systolic BP (mmHg)	149 ± 17	149 ± 17	149 ± 17
Daytime average diastolic BP (mmHg)	92 ± 12	90 ± 11	93 ± 13
Nighttime average systolic BP (mmHg)	130 ± 20	144 ± 18 *	121 ± 16
Nighttime average diastolic BP (mmHg)	80 ± 12	86 ± 10*	76 ± 12
Laboratory data			
Serum uric acid (mg/dL)	5.7 ± 1.6	6.0 ± 1.7 *	5.5 ± 1.5
High-sensitivity C-reactive protein (mg/dL)	0.08 ± 0.13	0.10 ± 0.15 *	0.07 ± 0.11
Low-density lipoprotein cholesterol (mg/dL)	116 ± 33	0.08 ± 0.13	0.08 ± 0.13
High-density lipoprotein cholesterol (mg/dL)	56 ± 15	52 ± 12 *	57 ± 16
Triglyceride (mg/dL)	97 ± 54	110 ± 60 *	90 ± 49
Fasting glucose (mg/dL)	96 ± 14	96 ± 14	96 ± 15
HOMA-IR	1.60 ± 1.84	1.73 ± 1.42	1.53 ± 2.04
HbA1c (%)	5.0 ± 0.5	5.1 ± 0.5	5.0 ± 0.4
Plasma renin activity (ng/mL/hr)	0.80 ± 0.67	0.75 ± 0.74	0.82 ± 0.63
Plasma aldosterone concentration (pg/mL)	74.2 ± 33.6	71.2 ± 37.5	75.9 ± 31.2
Epinephrine (pg/mL)	26.9 ± 18.8	25.5 ± 20.0	27.8 ± 18.2
Norepinephrine (pg/mL)	220 ± 87	200 ± 83	232 ± 88
Adiponectin (μg/mL)	10.1 ± 5.8	9.6 ± 5.4	10.4 ± 6.1
Leptin (μg/mL)	7.2 ± 6.5	8.0 ± 6.3	6.8 ± 6.6
Nitric oxide metabolites (μmol/L)	47.8 ± 31.1	41.4 ± 25.3 *	50.1 ± 31.3
Urinary data			
Urinary sodium (mEq/day)	191 ± 84	188 ± 83	195 ± 86
Urinary C-peptide (μg/day)	90 ± 53	92 ± 56	89 ± 52
Urinary epinephrine (μg/day)	11.4 ± 7.7	10.3 ± 8.6	12.0 ± 7.0
Urinary norepinephrine (μg/day)	162 ± 77	151 ± 70	169 ± 81
Urinary dopamine (μg/day)	1180 ± 1074	1058 ± 816	1242 ± 1188
Creatinine clearance (mL/min/1.73m^2^)	123 ± 36	121 ± 33	125 ± 37
Echocardiographic findings			
Left ventricular mass index (g/m^2^)	122 ± 35	130 ± 29 *	118 ± 37
Left ventricular ejection fraction (%)	66 ± 8	66 ± 8	67 ± 8
E/A ratio	0.95 ± 0.32	0.95 ± 0.33	0.96 ± 0.032

Data are expressed as mean ± SD or number. * *p* <0.05 vs. dipper. BP, blood pressure; HOMA-R, homeostasis model assessment ratio; E, early mitral inflow filling velocity; A, peak mitral filling velocity at atrial contraction.

**Table 2:** Factors associating with non-dipper status.

	Univariable Analysis		Multivariable Analysis	
Factor	Odds Ratio	*p* Value	Odd Ratio	*p* Value
Age	1.03 (1.00–1.06)	0.10		
Male	1.12 (0.58–2.18)	0.74		
Body mass index	1.10 (1.01–1.20)	<0.05 *	1.08 (0.98–1.20)	0.13
Alcohol intake	1.00 (0.99–1.02)	0.68		
Serum uric acid	1.25 (1.00–1.56)	<0.05 *	1.03 (1.00–1.05)	<0.05 *
High-sensitivity C-reactive protein	11.6 (0.67–201.0)	0.093	4.59 (0.23–90.2)	0.32
HOMA-R	1.06 (0.89–1.27)	0.51		
Plasma renin activity	0.85 (0.51–1.41)	0.52		
Plasma aldosterone concentration	0.96 (0.86–1.06)	0.40		
Epinephrine	0.99 (0.98–1.01)	0.48		
Norepinephrine	1.00 (1.00–1.00)	0.73		
Adiponectin	0.98 (0.92–1.03)	0.40		
Leptin	1.03 (0.98–1.08)	0.26		
Nitric oxide metabolites	0.99 (0.98–1.00)	0.070	0.99 (0.98–1.00)	0.15
Urinary sodium	1.00 (1.00–1.00)	0.66		
Urinary C-peptide	1.00 (0.99–1.01)	0.79		
Urinary epinephrine	0.97 (0.92–1.02)	0.21		
Urinary norepinephrine	1.00 (1.00–1.00)	0.98		
Urinary dopamine	1.00 (1.00–1.00)	0.33		
Creatinine clearance	1.00 (0.99–1.01)	0.57		

* *p* < 0.05 by logistic regression analysis. HOMA-R, homeostasis model assessment ratio.

**Table 3:** Association between nighttime systolic blood pressure and other clinical parameters in the non-dipper group.

	Univariable Analysis		Multivariable Analysis	
	Beta Value	*p* Value	Beta Value	*p* Value
Age	0.15	0.45		
Male	1.51	0.77		
Body mass index	0.98	0.093	0.55	0.38
Alcohol intake	0.10	0.37		
Serum uric acid	2.62	0.074	1.43	0.096
High-sensitivity C-reactive protein	36.21	<0.05 *	30.85	0.065
HOMA-R	−0.06	0.97		
Plasma renin activity	−3.73	0.27		
Plasma aldosterone concentration	−0.79	0.24		
Epinephrine	−0.02	0.55		
Norepinephrine	−0.04	0.24		
Adiponectin	−0.19	0.70		
Leptin	0.22	0.59		
Nitric oxide metabolites	−0.08	0.44		
Urinary sodium	0.03	0.36		
Urinary C-peptide	0.03	0.56		
Urinary epinephrine	0.23	0.45		
Urinary norepinephrine	0.01	0.92		
Urinary dopamine	0.00	0.95		
Creatinine clearance	−0.08	0.31		

* *p* < 0.05 by linear regression analysis. Multivariate R^2^-adjusted = 0.118, *p* = 0.034. HOMA-R; homeostasis model assessment ratio.

## Data Availability

Data are available upon appropriate request.
